# The Journal of Tehran University Heart Center Presented in the European Heart Journal

**Published:** 2018-10

**Authors:** Abbasali Karimi, Seyed Hesameddin Abbasi

**Affiliations:** *Tehran Heart Center, Tehran University of Medical Sciences, Tehran, Iran.*

We are delighted to inform you that the *Journal of Tehran University Heart Center* has recently been introduced in the* European Heart Journal *(Eur Heart J. 2018 Aug 1;39 (29):2697. doi:10.1093/eurheartj/ehy370). It is a source of pride to us that so high are the standards of the quality and validity of our journal that a well-known and prestigious journal like the *European Heart Journal*, which enjoys the highest impact factor (IF) among all cardiovascular journals in the world (2017 IF = 23.425), dedicates an entire page to the introduction of our publication to the wide spectrum of its readers, who may not be familiar with our scientific periodical. This success could not have been achieved without the sincere efforts and collaborations of our learned and distinguished International Editors, vastly experienced Editorial Board, highly motivated Advisory Board, very active and friendly Editorial Office, highly skilled Statistical Consultants, scholarly Reviewers, and various authors and researchers who choose our journal to submit their scientific works. We hereby acknowledge their invaluable contributions to this success.

**Figure:**
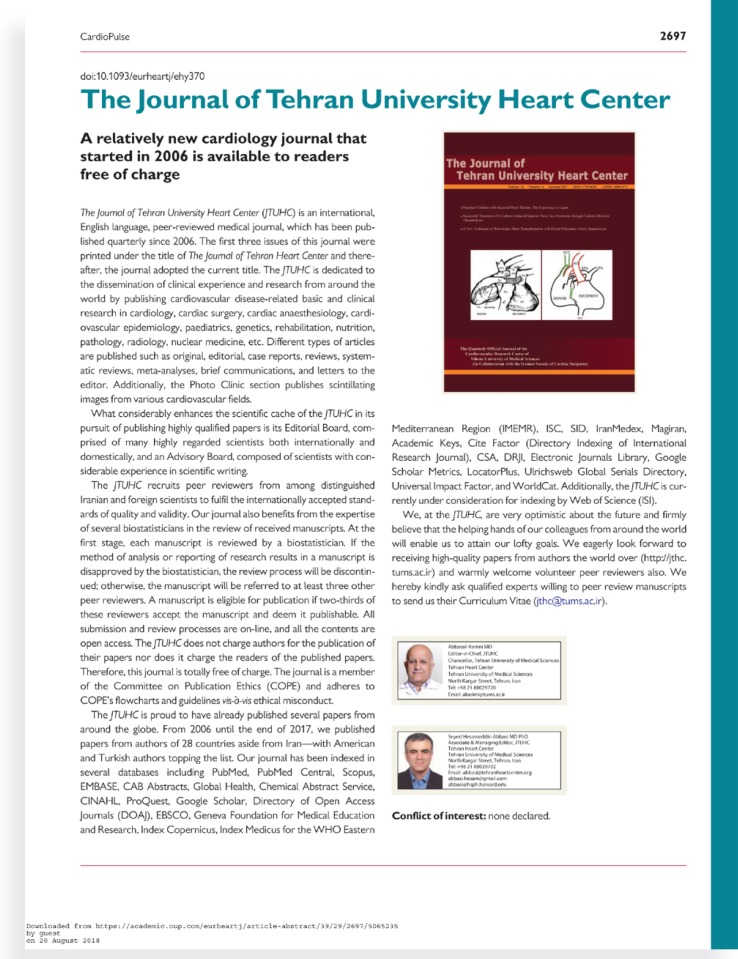


As the name of our medical periodical implies, the* Journal of Tehran University Heart Center* is supported by Tehran Heart Center and is the official journal of the Cardiovascular Research Center of Tehran University of Medical Sciences; however, we are honored that our publication has succeeded in providing a forum for a truly “international” exchange of information on all aspects of cardiovascular medicine. Indeed, we are proud to have published papers from such a wide array of countries from the world over as the United States, Turkey, United Kingdom, Spain, the Netherlands, Italy, India, Saudi Arabia, Belgium, Pakistan, Canada, Austria, Germany, Bahrain, Armenia, Poland, Croatia, Qatar, the United Arab Emirates, Greece, Morocco, Tunisia, Slovakia, Indonesia, and Nigeria.

We humbly encourage our readers to read the relevant paper in the *European Heart Journal*^1^ and we wish to take this opportunity to invite researchers willing to submit their manuscripts to our publication to meticulously read our journal’s editorial, entitled “Scientific authors should be more careful”.^2^ Our contributors’ taking heed of those points prior to the submission of their manuscripts will enable us to maintain the high quality of the published papers.
